# Effect of *Morus alba* leaf extract dose on lipid oxidation, microbiological stability, and sensory evaluation of functional liver pâtés during refrigerated storage

**DOI:** 10.1371/journal.pone.0260030

**Published:** 2021-12-23

**Authors:** Agnieszka Bilska

**Affiliations:** Department of Meat Technology, Poznan University of Life Sciences, Poznan, Poland; Bahauddin Zakariya University, PAKISTAN

## Abstract

Mulberry (*Morus alba L*.), and above all the extract from the leaves of this plant, is a natural medicine that has been used in traditional medicine for hundreds of years. Mulberry leaves contains polyphenol compounds: flavonoids, coumarins, numerous phenolic acids, as well as terpenes and steroids. The antioxidant effect of these compounds may be beneficial to the fat fraction of meat products, thereby increasing their functional qualities. The aim of the study was to evaluate the effectiveness of the use of mulberry water leaf extract, as an additive limiting adverse fat changes and affecting the functionality in model liver pâtés. Pork pâtés were prepared by replacing 20% of animal fat with rapeseed oil (RO), and water extract of mulberry leaves was added in the proportion of 0.2%, 0.6% and 1.0%. It has been shown that the addition of mulberry leaf extract delayed the appearance of primary and secondary fat oxidation products. The most effective antioxidant effect during 15-day storage was observed in the sample with the addition of 0.6% and 1.0% water mulberry leaf extract. These samples also showed inhibiting activity against angiotensin-converting enzymes and cholinesterase’s. During storage, the tested pâtés had a high sensory quality with unchanged microbiological quality. Mulberry leaf extract can be an interesting addition to the production of fat meat products, delaying adverse changes in the lipid fraction and increasing the functionality of products.

## 1. Introduction

White mulberry (*Morus alba* L.) originates from China, Japan, and India. There are reports in the literature about the beneficial effects of mulberry leaf and fruit preparations in such diseases as diabetes, hypertension, obesity, Parkinson’s disease, neurodegenerative diseases, atherosclerosis or cancer [1, 2]. This effect is due to the presence of many compounds with biological activity. Mulberry contains polyphenol compounds, including flavonoids, e.g., quercetin 3-(6-malonylglycoside), kaempferol 3- (6-malonylglycoside), rutin, isoquercetin, astragaline, moracetin derivatives and other glycosides. Mulberry is also a source of tannins and coumarins (scopol and scimine). The presence of numerous phenolic acids (chlorogenic acid, caffeic acid, hydroxybenzoic acid or ferulic acid) was determined in mulberry leaves and fruits. In addition, mulberry was shown to contain terpenes such as citral, linalool acetate, linalool or cis-3-hexene-1-ol, and steroids, e.g. β-sitosterol. Important pharmacologically active ingredients of mulberry are alkaloids that may affect carbohydrate metabolism: 1,5-dideoxy-1,5-imino-D-sorbitol (DNJ) and its derivatives Niidome et al. [3].

Mulberry compounds can beneficially act as inhibitors of enzymes such as angiotensin-converting enzyme or cholinesterase. Kojima et al. [4] confirmed the effect of methanolic mulberry leaf extract on acetylcholine esterase inhibiting activity. It’s repetitive Kang et al. [5] confirmed the activity of mulberry leaf preparations and active compounds contained in them against neurological disorders such as Alzheimer’s disease and Parkinson’s disease. Niidome et al. [3] also confirmed the neuroprotective effect of mulberry leaves as raw materials rich in compounds that inhibit β-amyloid formation.

Recently in many scientific reports, attention has been paid to the beneficial properties of mulberry and the possibility of its application in the production of foodstuffs. The use of this raw material in food technology applies to both leaves and fruits. Fruits are currently used in the production of juices, jams, marmalades, wines and even ice cream. Mulberry fruits can be used as an ingredient in muesli [6]. Leaves, on the other hand, are a component of teas, cakes and breads [7].

There was mention several times in the literature about the antioxidant activity of polyphenolic compounds against fat fractions of meat products. In regards of this, applications in pâtés, e.g. rosemary [8] or ginkgo [9], are known. Flavonoids have the ability to inactivate superoxide, peroxide and hydroxyl radicals as well as oxygen *in statu nascendi*. In addition, polyphenols can form complexes with metals that catalyze the oxidation reaction and inhibit the activity of oxidizing enzymes such as lipoxygenases. The antioxidant effect is also demonstrated by mulberry fruits and leaves, which is related to the content of: ascorbic acid (in amounts of approx. 10–30 mg / 100 g), polyphenols (mainly flavonoids, anthocyanins and resveratrol), steroids and terpenes [1, 10]. In the production of food of meat origin, in addition to positive sensory sensations, its health state is an important element. Therefore, there are trends towards the use of natural additives, which may also be beneficial to consumer health. Hence, the aim of the study was to evaluate the effectiveness of mulberry water leaf extract as an additive limiting adverse fat changes and affecting the functionality in model meat pâtés.

## 2. Materials and methods

### 2.1. Materials

#### 2.1.1. Mulberry extract

The material used for the study was a water extract from the leaves of white mulberry *Morus alba* L, of the cultivar Wielkolistna Żółwińska. Leaves were collected at the plantation of the Institute of Natural Fibers and Medicinal Plants in Poznan, at the Petkowo Experimental Department. Leaves milled in a grinder (Retsch GmbH, Germany; Retsch GM200 at 5000 r/s, pulsating for 10 s) were subjected to water extraction using the three-time extraction method. A weighted amount of leaf powder (50 g) was poured over with a total of 1000 ml of water at a temperature of 85°C and extracted each time for 4 minutes. The extraction was repeated three times. Each time the extract was filtered, centrifuged (2697 x g, 15 min). Each fraction was decanted and filtered (Whatman 1:11 μm). The supernatants were combined and lyophilized. Extraction efficiency was 6.92%, total polyphenol content 151 mg/g calculated as chlorogenic acid.

#### 2.1.2. Production process of liver pâté

Liver pâtés were produced at the Experimental Processing Plant of the Department of Meat Technology of the Poznan University of Life Sciences. The products were prepared according to the following recipe: class II pork meat– 43%, fine fat– 42%, liver– 15% and broth in the proportion of 30% in relation to the meat and fat raw material. While slicing, a mixture of spices was added in the following amount (per 1 kg of forcemeat): salt– 1.5%; pepper– 0.15%; marjoram– 0.05% and onions– 0.4%. This raw material composition was the reference product for experimental variants. Sausages were also produced in which 20% of animal fat was replaced with rapeseed oil (RO), and mulberry leaf water extract was added (Table 1).

**Table 1:** Formulations of liver pâté [g kg^-1^].

Ingredients [g/kg]	Sample				
	Control	20% RO replacement	20% RO replacement + 0.2% mulberry	20% RO replacement + 0.6% mulberry	20% RO replacement + 1.0% mulberry
Pork meat (class II)	430	430	430	430	430
Pork back fat	420	336	336	336	336
Rapeseed oil (RO)	-	84	84	84	84
Pork liver	150	150	150	150	150
Mix of spices	21	21	21	21	21
Mulberry leaf water extract	-	-	2	6	10

The production process of experimental sausages: meat and fat raw material after cooking to a semi-soft state, was sliced, oil was added. In the recipe spices and mulberry extract provided for. The pre-cuttered liver was dosed at the end of the slicing process. The final temperature of the forcemeat was 40°C. Semi-permeable collagen casings with a diameter of 40 mm were stuffed with forcemeat and subsequently poached to 70°C and cooled to 4°C. Finished products were placed in a cold room at approx. 4°C. Samples for testing were taken on days 1, 5, 8, 12 and 15 after production.

### 2.2. Determination of primary and secondary fat oxidation products

#### 2.2.1. Peroxide value (PV)

Determination of peroxide value (PV) was carried out in accordance with the ISO standard [11]. Extraction of the lipid fraction was carried out according to the procedure of Folch et al. [12] (chloroform:methanol solvent ratio of 2:1, v/v).

The results are expressed as milliequivalents of active oxygen/kg sample (meq. O_2_ x kg^1^).

#### 2.2.2. Malonaldehyde content by TBARS

The value of the TBARS index (substances reacting with 2-thiobarbituric acid) was determined by a distillation method of Tarladgis et al. [13], modified by Pikul et al. [14].

The results are expressed as mg malonaldehyde/kg samples (mg MDA x kg^-1^).

#### 2.3. Determination of activity as a cholinesterase inhibitor (ChE)

Inhibition of cholinesterases was determined in water extracts obtained from experimental sausages. They were prepared after previous defatting (with chloroform) by shaking for 30 minutes at 18°C.

Determination of extract activities as acetylcholinesterase (AChE) and butyrylcholinesterase (BChE) inhibitors was performed using a modified spectrometric method [15] described by Kobus-Cisowska et al. [16]. The measurements were carried out using a POLARstar Omega plate reader (BMG LABTECH) in 96-well plates with a maximum volume of 300 μl. The color reaction of acetylthiocholine/butyrylthiocholine hydrolysis was measured at 412 nm for 10 minutes after enzyme application to the microplate.

Reagent solutions were prepared in Tris-HCl buffer (50 mmol/dm^3^, pH 8.0). Enzyme solutions were prepared by dissolving 2 U/ml in 2 ml phosphate buffer. The composition of the reaction mixture was as follows: 0.035 cm^3^ of the analyzed sample, 0.086 cm^3^ Tris-HCl buffer (50 mmol/dm^3^, pH 8), 0.035 cm^3^ ATChI or BTCh (1.5 mmol/dm^3^), 0.194 cm^3^ DTNB (0.3 mmol/dm^3^ with the addition of 10 mmol/dm^3^ NaCl and 2 mmol/dm^3^ MgCl_2_ x 6 H_2_O), AChE lub BChE solution. The measurement was made 15 min (BChE) or 30 min (AChE) after application to the microplate. At the same time, a positive control containing the known ChE inhibitor–eserine, and negative control without a ChE inhibitor, was analyzed, and the background of the analyzed samples was considered (n = 5).

Anti-ChE activity was calculated using eserine standard curves in the concentration range: 0.08 μmol/dm^3^–6.50 μmol/dm^3^ (AChE) and 0.08 μmol/dm^3^–8.30 μmol/dm^3^ (BChE). The results are expressed in eserine equivalents (μM eserine/g dw extract).

### 2.4. Determination of activity against angiotensin-converting enzyme I

Determination of the activity against ACE was carried out according to the methodology of Cushman and Cheung [17] with modifications. The determination consisted in assessing the degree of inhibition of the analyzed experimental sausages against angiotensin-converting enzyme I (ACE). The method was based on the breakdown of hippuryl-L-histidyl-L-leucine (HHL) substrate to hypuric acid (HA) and histidine-leucine (HL) under the action of angiotensin-converting enzyme I. Then, the degree of HA release from HHL was directly related to the ACE activity. Inhibition was measured spectrophotometrically using a plate reader (POLARstar Omega) at a wavelength of 492 nm. The results are given as a percentage of inhibition.

### 2.5. Microbiological analysis

The total number of microorganisms was determined according to the guidelines contained in the standard: ISO 4833–1:2013 [18]. Microbiological examination to determine the count of enterococci was performed according to the standard: PN-A-82055-7:1997 [19] on Slanetz and Bartley medium (BTL Ltd., Poland).

Isolation and determination of the number of bacilli from the family *Enterobacteriaceae* was carried out in accordance with the guidelines present in the standards: ISO 21528–1:2017 [20] and ISO 21528–2:2017 [21]. A medium with bile, neutral red, crystal violet and glucose (VRBG) was used for the determination. Agar medium with cetrimide, fucidin and cephaloridine (CFC) was used for the detection, isolation and determination the bacterial count of the genus *Pseudomonas* (BTL Ltd., Poland). The determination was carried out in accordance with the guidelines contained in the standard ISO 13720:2010 [22].

### 2.6. Measurement of pH and water activity a_w_

Active acidity (pH) was assessed by the method according to the ISO standard [23]. Water activity (a_w_) was measured using an AquaLab series 4TE apparatus (Pullman, USA) according to the camera’s instruction manual.

### 2.7. Sensory quality assessment

Sensory evaluation was performed based on the guidelines contained in the following standards: ISO 5492:2008 and ISO 13299:2016 [24, 25].

Sensory quality assessment was conducted by a 20-persons well trained team, with proven sensory sensitivity. The scaling method was applied, using an unstructured graphic scale, with appropriate boundary determination, in which a 10-cm segment was the scale. The obtained results were replaced by numerical values expressed in conventional units (points). Nine sensory characteristics were measured to quantify sensory quality: 4 for odour, 4 for taste, and 1 for overall desirability. For the odour and taste discriminants, edge markings were used: undetectable– 0, intense– 10, for overall desirability they were the markings: undesirable– 0; desirable– 10.

### 2.8. Statistical analysis

The obtained research results were subjected to statistical analysis using the STATISTICA 13.1 software and Excel 2010.

The results presented in this work, are arithmetic means of two experimental series and three replications. The average values of the examined characteristics were compared using the analysis of variance for factorial design, and Tukey’s test assessed the intergroup differences. Statistical inference was carried out at the significance level of α = 0.05.

Correlation between the variables was tested using a linear regression analysis y = Ax+B, where y–dependent variable (tested parameter value), x—independent variable (sample type, storage time), A–coefficient with an independent variable (angle tangent of curve slope), B–free term. Statistical analysis of changes in the regression angle (A/24 h coefficient) allowed determining the dynamics of occurring changes. In addition, the principal component analysis (PCA) method was used.

## 3. Results

### 3.1. Determination of primary and secondary fat oxidation products in experimental sausages

The peroxide value in samples with 20% animal fat replacement with rapeseed oil increased until day 12 after production (Table 2). Further storage of the tested sausages resulted in a statistically significant decrease in its value. The smallest difference in peroxide value between day 1 and 15 after production was found in the sample with the addition of 0.6% mulberry extract (Δ = 0.07). Analyzing the values of regression coefficients was found that the process of peroxide formation was inhibited most effectively by the addition of 0.6% and 1.0% mulberry leaf extract. In the present experiment, the highest values of TBARS index were recorded for control and 20% animal fat replacement with rapeseed oil samples throughout the storage period (Table 2). The changes that have occurred in the peroxide content during product storage, increased dynamics of secondary lipid oxidation products in samples with 20% substitution of fine animal fat with rapeseed oil, and were lower in comparison to the control sample.

**Table 2:** The influence of storage time on changes in fats of the experimental sausages $\bar{x}$ (n = 6) ±sd.

Type of sample	Storage time (days)					Ax10^-3^/24h coeff.	R^2^	Δ
	1	5	8	12	15			
Peroxide value (mEq O_2_/kg of sample) LSD A = 0.01; LSD B = 0.01								
Control sample	0.20^a^^A^ ± 0.00	0.22^b^^B^ ± 0.01	0.27^b^^cC^ ± 0.03	0.44^eE^ ± 0.01	0.36^dD^ ± 0.01	16	0.75	0.16
20% RO replacement	0.19^a^^A^ ± 0.03	0.20^a^^A^ ± 0.02	0.30^d^^B^ ± 0.05	0.42^dD^ ± 0.05	0.35^cdC^ ± 0.02	16	0.77	0.16
20% RO replacement + 0.2% mulberry	0.19^a^^A^ ±0.01	0.21^a^^b^^B^ ± 0.01	0.33^eC^ ± 0.01	0.39^cD^ ± 0.02	0.38^eD^ ± 0.02	16	0.88	0.19
20% RO replacement + 0.6% mulberry	0.19^a^^A^ ± 0.00	0.20^a^^A^ ± 0.01	0.28^cC^ ± 0.01	0.33^b^^D^ ± 0.01	0.26^a^^B^ ± 0.02	8	0.55	0.07
20% RO replacement + 1.0% mulberry	0.19^a^^A^ ± 0.01	0.20^a^^A^ ± 0.01	0.25^a^^B^ ± 0.01	0.29^a^^C^ ± 0.01	0.28^b^^C^ ± 0.01	8	0.88	0.09
TBARS values (mg/kg of sample) LSD A = 0.01; LSD B = 0.01								
Control sample	1.18^e^^A^ ± 0.01	1.49^e^^B^ ± 0.02	1.55^eC^ ± 0.02	1.63^eD^ ± 0.02	1.87^eE^ ± 0.05	43	0.93	0.69
20% RO replacement	0.98^c^^A^ ± 0.02	1.25^d^^B^ ± 0.02	1.34^dC^ ± 0.02	1.45^dD^ ± 0.02	1.79^dE^ ± 0.01	52	0.94	0.81
20% RO replacement + 0.2% mulberry	0.90^b^^A^ ± 0.01	1.14^c^^B^ ± 0.01	1.27^cC^ ± 0.01	1.30^cD^ ± 0.01	1.45^cE^ ± 0.02	36	0.93	0.55
20% RO replacement + 0.6% mulberry	1.02^d^^A^ ± 0.04	1.09^b^^B^ ± 0.01	1.12^b^^C^ ± 0.01	1.13^b^^C^ ± 0.01	1.19^bD^ ± 0.02	11	0.94	0.17
20% RO replacement + 1.0% mulberry	0.88^a^^A^ ± 0.03	1.04^a^^B^ ± 0.01	1.06^a^^C^ ± 0.02	1.11^a^^D^ ± 0.01	1.16^a^^E^ ± 0.01	18	0.90	0.28

RO–rapeseed oil.

Δ–difference between the results of assessment on the 15^th^ and 1^st^ day after production.

$\bar{x}$—mean value, n–number of replications, sd–standard deviation.

LSD A–the least significant difference for the type of sample.

LSD B–the least significant difference for storage time.

a, b …–mean values ± standard deviation followed by different letters in rows refer to statistically significant differences between sample types (p ≤ 0.05).

A, B …–mean values ± standard deviation followed by different letters in columns refer to statistically significant differences in storage time (p ≤ 0.05).

Linear regression equation: y = Ax+B (y—dependent variable, x—independent variable, A—independent variable coefficient per line slope. B—intercept), coefficient A/24h –change in coefficient A during 24h-storage, R^2^– coefficient of determination, p < 0.05.

Measurements of the content of secondary oxidation reaction products showed that the addition of 0.6% and 1.0% mulberry leaf extract effectively slowed down the formation of secondary fat oxidation products. The lowest difference in malonaldehyde content in the tested sausages, between day 1 and 15 after production, was found in the samples with the addition of 0.6% mulberry extract (0.17 mg MDA/kg sample). Directional coefficients in regression equations shows the relationships between storage time, the addition of antioxidants and the content of secondary oxidation products. Analyzing the obtained coefficients, was found that 0.6% water mulberry leaf extract showed the most effective protective effect in experimental sausages.

### 3.2. Evaluation of the activity of water mulberry leaf extracts in inhibiting cholinesterase activity in experimental sausages

The study evaluated the effect of the addition of water mulberry leaf extract on health-promoting properties of liver pâté in terms of inhibition of cholinesterases–acetylcholinesterase (AChE) and butyrylcholinesterase (BChE) activity (Table 3). Based on a two-factorial analysis of variance, storage time had no significant effect on acetylcholinesterase activity. On the other hand, sample type, i.e., the substitution of animal fat with rapeseed oil in a proportion of 20%, and the addition of mulberry leaf extract had a statistically, significant impact on AChE activity. The addition of mulberry leaf extract statistically, significantly increased acetylcholinesterase inhibition. Directional coefficient values for variants with the addition of mulberry were higher than the control sample, which indicated the inhibitory activity of the applied extract. The samples showed BChE activity through the entire storage period. The highest degree of butyrylcholinesterase inhibition was found in cold cut with 1.0% addition of water mulberry leaf extract. The lower percentages of water mulberry leaf extract showed less BChE inhibitory activity. It was also found that the addition of water mulberry extract to the experimental cold cuts had a greater impact on the inhibition of AChE than BChE.

**Table 3:** The influence of water mulberry leaf extract on AChE, BChE and angiotensin-converting enzyme activity in experimental sausages $\bar{x}$ (n = 4) ±sd.

Type of sample	Storage time (days)					Ax10^-3^/24h coeff.	R^2^
	1	5	8	12	15		
AChE inhibitory activity (μM eserine/g s.m.) LSD A = 0.010; LSD B = 0.009							
Control sample	0.059^a^^A^ ± 0.011	0.056^a^^A^ ± 0.005	0.056^a^^A^ ± 0.004	0.057^a^^A^ ± 0.004	0.052^a^^A^ ± 0.002	-0.37	0.63
20% RO replacement	0.060^a^^A^ ± 0.007	0.064^a^^b^^A^ ± 0.006	0.055^a^^A^ ± 0.002	0.060^a^^A^ ± 0.012	0.061^a^^A^ ± 0.005	0.19	0.08
20% RO replacement + 0.2% mulberry	0.076^b^^A^^B^ ± 0.006	0.073^b^^A^^B^ ± 0.007	0.072^b^^A^^B^ ± 0.007	0.071^b^^A^ ± 0.019	0.081^b^^B^ ± 0.004	0.21	0.08
20% RO replacement + 0.6% mulberry	0.113^c^^B^ ± 0.013	0.094^c^^A^ ± 0.002	0.094^c^^A^ ± 0.012	0.091^c^^A^ ± 0.012	0.092^c^^A^ ± 0.002	-1.32	0.64
20% RO replacement + 1.0% mulberry	0.161^dD^ ± 0.010	0.151^dC^ ± 0.002	0.145^dC^ ± 0.015	0.131^d^^B^ ± 0.008	0.122^d^^A^ ± 0.013	-2.80	0.99
BChE inhibitory activity (μM eserine/g s.m.) LSD A = 0.003; LSD B = 0.002							
Control sample	0.002^a^^A^ ± 0.001	0.004^a^^A^ ± 0.000	0.004^a^^A^ ± 0.000	0.004^a^^A^ ± 0.001	0.003^a^^A^ ± 0.001	0.04	0.09
20% RO replacement	0.002^a^^A^ ± 0.001	0.003^a^ ± 0.000	0.004^a^^A^ ± 0.001	0.004^a^^A^ ± 0.000	0.004^a^^A^ ± 0.000	0.14	0.80
20% RO replacement + 0.2% mulberry	0.011^b^^A^^B^ ± 0.001	0.011^b^^A^^B^ ± 0.001	0.012^b^^B^ ± 0.002	0.009^b^^A^ ± 0.002	0.010^b^^A^^B^ ± 0.003	-0.11	0.34
20% RO replacement + 0.6% mulberry	0.014^bC^ ± 0.001	0.013^b^^B^^C^ ± 0.001	0.010^b^^A^ ± 0.000	0.011^b^^A^^B^ ± 0.001	0.010^b^^A^ ± 0.001	-0.27	0.72
20% RO replacement + 1.0% mulberry	0.019^c^^B^ ± 0.001	0.018^c^^B^ ± 0.002	0.018^c^^B^ ± 0.001	0.012^b^^A^ ± 0.001	0.011^b^^A^ ± 0.001	-0.63	0.86
Angiotensin-converting enzyme activity (% inhibition) LSD A = 0.21; LSD B = 0.19							
Control sample	0.50^a^^A^ ± 0.03	0.54^a^^A^ ± 0.10	0.63^a^^A^ ± 0.10	0.57^a^^A^ ± 0.00	0.57^a^^A^ ± 0.02	4.76	0.31
20% RO replacement	0.55^a^^A^ ± 0.21	0.56^a^^A^ ± 0.07	0.54^a^^A^ ± 0.09	0.51^a^^A^ ± 0.03	0.52^a^^A^ ± 0.03	-2.69	0.61
20% RO replacement + 0.2% mulberry	1.99^b^^B^ ± 0.10	1.91^b^^B^ ± 0.08	1.52^b^^A^ ± 0.05	1.51^b^^A^ ± 0.09	1.58^b^^A^ ± 0.12	-34.84	0.70
20% RO replacement + 0.6% mulberry	2.82^c^^B^ ± 0.31	2.52^c^^A^ ± 0.24	2.47^c^^A^ ± 0.41	2.46^c^^A^ ± 0.11	2.45^c^^A^ ± 0.15	-23.17	0.68
20% RO replacement + 1.0% mulberry	4.32^d^^B^ ± 0.32	3.41^d^^A^ ± 0.11	3.48^d^^A^ ± 0.26	3.41^d^^A^ ± 0.21	3.48^d^^A^ ± 0.37	-49.50	0.49

RO–rapeseed oil.

Δ–difference between the results of assessment on the 15^th^ and 1^st^ day after production.

$\bar{x}$—mean value, n–number of replications, sd–standard deviation.

LSD A–the least significant difference for the type of sample.

LSD B–the least significant difference for storage time.

a, b …–mean values ± standard deviation followed by different letters in rows refer to statistically significant differences between sample types (p ≤ 0.05).

A, B …–mean values ± standard deviation followed by different letters in columns refer to statistically significant differences in storage time (p ≤ 0.05).

Linear regression equation: y = Ax+B (y—dependent variable, x—independent variable, A—independent variable coefficient per line slope. B—intercept), coefficient A/24h –change in coefficient A during 24h-storage, R^2^– coefficient of determination, p < 0.05.

Liver pâté produced with the addition of mulberry leaf extracts had angiotensin-converting enzyme I inhibitory activity. Among the tested samples, the activity of water mulberry leaf extract was demonstrated, which was dependent on the amount of extract added during the production of experimental sausages.

### 3.3. The effect of the addition of mulberry leaf water extract on the microbiological safety of liver pâté

During storage of the tested sausages, the lowest increase in aerobic bacteria was found in samples with the addition of 0.6% and 1.0% mulberry water leaf extract. In addition, it was noted that the content of aerobic microorganisms in the tested samples did not exceed 2.65log_10_cfu/g during 15 days of storage (Table 4). The number of *Pseudomonas* bacteria was determined at 10^2^−10^3^ cfu/g. The smallest increase was found in the sample with 20% animal fat replacement with rapeseed oil and in the sample in which, in addition to fat replacement, 0.6% mulberry leaf water extract was added.

**Table 4:** The influence of storage time on changes in microbial quality and water activity in experimental sausages $\bar{x}$ (n = 6)±sd.

Type of sample	Storage time (days)					Ax10^-3^/24h coeff.	R^2^
	1	5	8	12	15		
Total count of mesophilic bacteria (log_10_ cfu/g) LSD A = 0.06; LSD B = 0.06; LSD A*B = 0.13							
Control sample	1.44^a^^A^ ± 0.36	2.18^d^^B^ ± 0.07	2.25^a^^C^ ± 0.02	2.39^a^^D^ ± 0.05	2.55^a^^b^^E^ ± 0.05	70	0.83
20% RO replacement	1.60^b^^A^ ± 0.09	2.07^c^^B^ ± 0.06	2.28^a^^C^ ± 0.04	2.38^a^^D^ ± 0.02	2.64^cE^ ± 0.06	68	0.94
20% RO replacement + 0.2%mulberry	1.72^c^^A^ ± 0.04	1.81^b^^B^ ± 0.04	2.24^a^^C^ ± 0.04	2.46^b^^D^ ± 0.01	2.61^b^^cE^ ± 0.01	69	0.95
20% RO replacement + 0.6%mulberry	1.70^c^^A^ ± 0.16	1.70^a^^A^ ± 0.07	2.25^a^^B^ ± 0.04	2.39^a^^C^ ± 0.02	2.56^a^^b^^D^ ± 0.03	68	0.89
20% RO replacement + 1.0%mulberry	1.70^c^^A^ ± 0.07	1.83^b^^B^ ± 0.03	2.26^a^^C^ ± 0.04	2.44^a^^b^^D^ ± 0.03	2.53^a^^E^ ± 0.01	65	0.94
*Pseudomonas* (log_10_ cfu/g) LSD A = 0.05; LSD B = 0.05; LSD A*B = 0.10							
Control sample	1.58^a^^b^^A^ ± 0.19	2.07^c^^B^ ± 0.04	2.40^dC^ ± 0.02	2.54^dD^ ± 0.01	2.70^cE^ ± 0.05	78	0.94
20% RO replacement	1.57^a^^b^^A^ ± 0.06	2.05^c^^B^ ± 0.06	2.11^b^^C^ ± 0.03	2.21^b^^D^ ± 0.03	2.42^a^^E^ ± 0.06	54	0.90
20% RO replacement + 0.2% mulberry	1.57^a^^b^^A^ ± 0.06	1.68^b^^B^ ± 0.05	2.01^a^^C^ ± 0.04	2.11^a^^D^ ± 0.03	2.52^b^^E^ ± 0.02	66	0.93
20% RO replacement + 0.6% mulberry	1.56^a^^b^^A^ ± 0.11	1.71^b^^B^ ± 0.10	2.22^cC^ ± 0.04	2.31^cD^ ± 0.04	2.45^a^^E^ ± 0.02	67	0.91
20% RO replacement + 1.0% mulberry	1.52^a^^A^ ± 0.17	1.60^a^^B^ ± 0.09	2.17^cC^ ± 0.04	2.29^cD^ ± 0.05	2.53^b^^E^ ± 0.04	77	0.92
Water activity LSD A = 0.00; LSD B = 0.00; LSD A*B = 0.00							
Control sample	0.98^b^^E^ ± 0.00	0.96^a^^D^ ± 0.00	0.95^a^^C^ ± 0.00	0.95^a^^B^ ± 0.00	0.93^a^^A^ ± 0.00	-3.13	0.91
20% RO replacement	0.97^a^^D^ ± 0.00	0.96^a^^b^^C^ ± 0.00	0.96^b^^C^ ± 0.00	0.95^a^^B^ ± 0.00	0.94^b^^A^ ± 0.00	-2.00	0.95
20% RO replacement + 0.2% mulberry	0.97^a^^C^ ± 0.00	0.97^cC^ ± 0.00	0.96^b^^B^ ± 0.00	0.96^b^^B^ ± 0.01	0.95^c^^A^ ± 0.00	-1.40	0.86
20% RO replacement + 0.6% mulberry	0.97^a^^C^ ± 0.00	0.96^c^^B^ ± 0.00	0.96^b^^B^ ± 0.00	0.96^b^^B^ ± 0.00	0.96^d^^A^ ± 0.00	-0.59	0.53
20% RO replacement + 1.0% mulberry	0.97^a^^C^ ± 0.00	0.97^cC^ ± 0.00	0.96^b^^B^ ± 0.00	0.96^b^^B^ ± 0.00	0.96^e^^A^ ± 0.00	-0.85	0.73
pH LSD A = 0.03; LSD B = 0.03; LSD A*B = 0.08							
Control sample	6.43^a^^A^ ± 0.03	6.42^b^^A^ ± 0.01	6.46^c^^A^ ± 0.01	6.50^b^^B^ ± 0.03	6.56^a^^b^^C^ ± 0.04	9.59	0.87
20% RO replacement	6.50^c^^B^ ± 0.01	6.46^c^^A^ ± 0.02	6.46^c^^A^ ± 0.01	6.50^b^^B^ ± 0.03	6.57^b^^C^ ± 0.01	4.98	0.38
20% RO replacement + 0.2% mulberry	6.49^b^^C^^B^ ± 0.23	6.43^b^^C^^A^ ± 0.02	6.42^b^^A^ ± 0.03	6.48^b^^B^ ± 0.04	6.56^a^^b^^C^ ± 0.02	5.24	0.27
20% RO replacement + 0.6% mulberry	6.44^a^^B^ ± 0.08	6.36^a^^A^ ± 0.01	6.36^a^^A^ ± 0.02	6.44^a^^B^ ± 0.04	6.55^a^^b^^C^ ± 0.01	8.26	0.35
20% RO replacement + 1.0% mulberry	6.43^a^^B^ ± 0.08	6.38^a^^A^ ± 0.01	6.38^a^^A^ ± 0.01	6.43^a^^B^ ± 0.01	6.53^a^^C^ ± 0.01	7.21	0.43

RO–rapeseed oil.

Δ–difference between the results of assessment on the 15^th^ and 1^st^ day after production.

$\bar{x}$—mean value, n–number of replications, sd–standard deviation.

LSD A–the least significant difference for the type of sample.

LSD B–the least significant difference for storage time.

a, b …–mean values ± standard deviation followed by different letters in rows refer to statistically significant differences between sample types (p ≤ 0.05).

A, B …–mean values ± standard deviation followed by different letters in columns refer to statistically significant differences in storage time (p ≤ 0.05).

Linear regression equation: y = Ax+B (y—dependent variable, x—independent variable, A—independent variable coefficient per line slope. B—intercept), coefficient A/24h –change in coefficient A during 24h-storage, R^2^– coefficient of determination, p < 0.05.

No heat-resistant enterococci were found in experimental sausages during 15 days of storage. No bacteria from the family *Enterobacteriaceae* were found either.

The water activity of experimental sausages ranged from 0.93 (for the control sample on the 15th day after production) to 0.98 (directly after production also for the control sample). The lowest changes in water activity during storage were observed for the sample in which, in addition to exchanging 20% of animal fat for rapeseed oil, 0.6% and 1.0% mulberry leaf water extract was added.

A similar course of pH variation characterized the experimental cold cuts during the 15-d storage (Table 4).

### 3.4. The effect of the addition of mulberry water leaf extract on the sensory quality of experimental sausages

**Fig 1:**
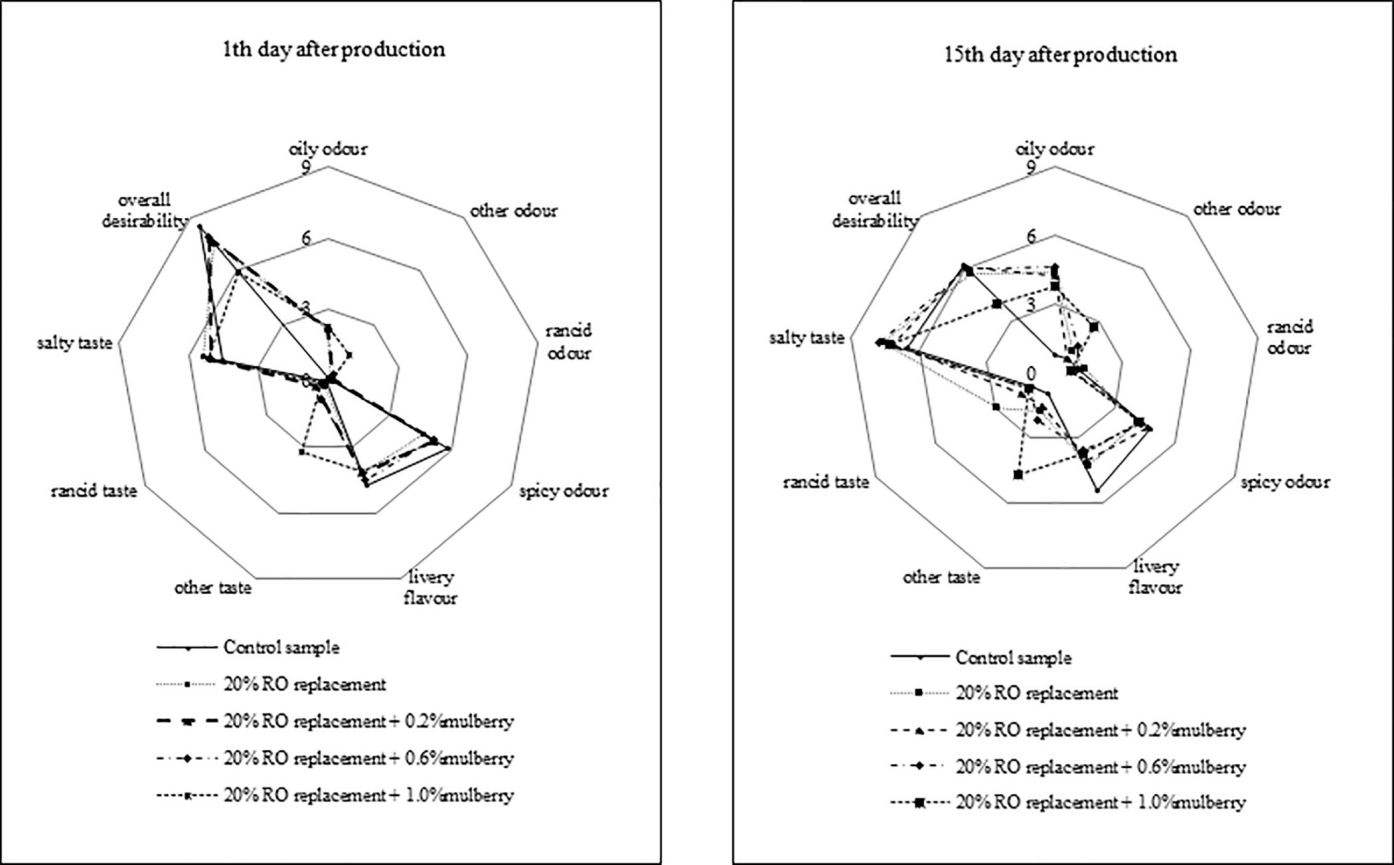
Sensory evaluation of experimental sausages.

The control sample showed the highest overall desirability on the first and fifteenth day after production (Fig 1). In the smell and taste profile, no negative effect of the addition of mulberry leaf water extract in the proportion of 0.2% and 0.6% on the examined parameters was found. On the other hand, the addition of 1.0% of this extract caused the appearance of a foreign smell and taste. The evaluation team described it as grassy/dried grass.

### 3.5. Principal component analysis (PCA)

**Fig 2:**
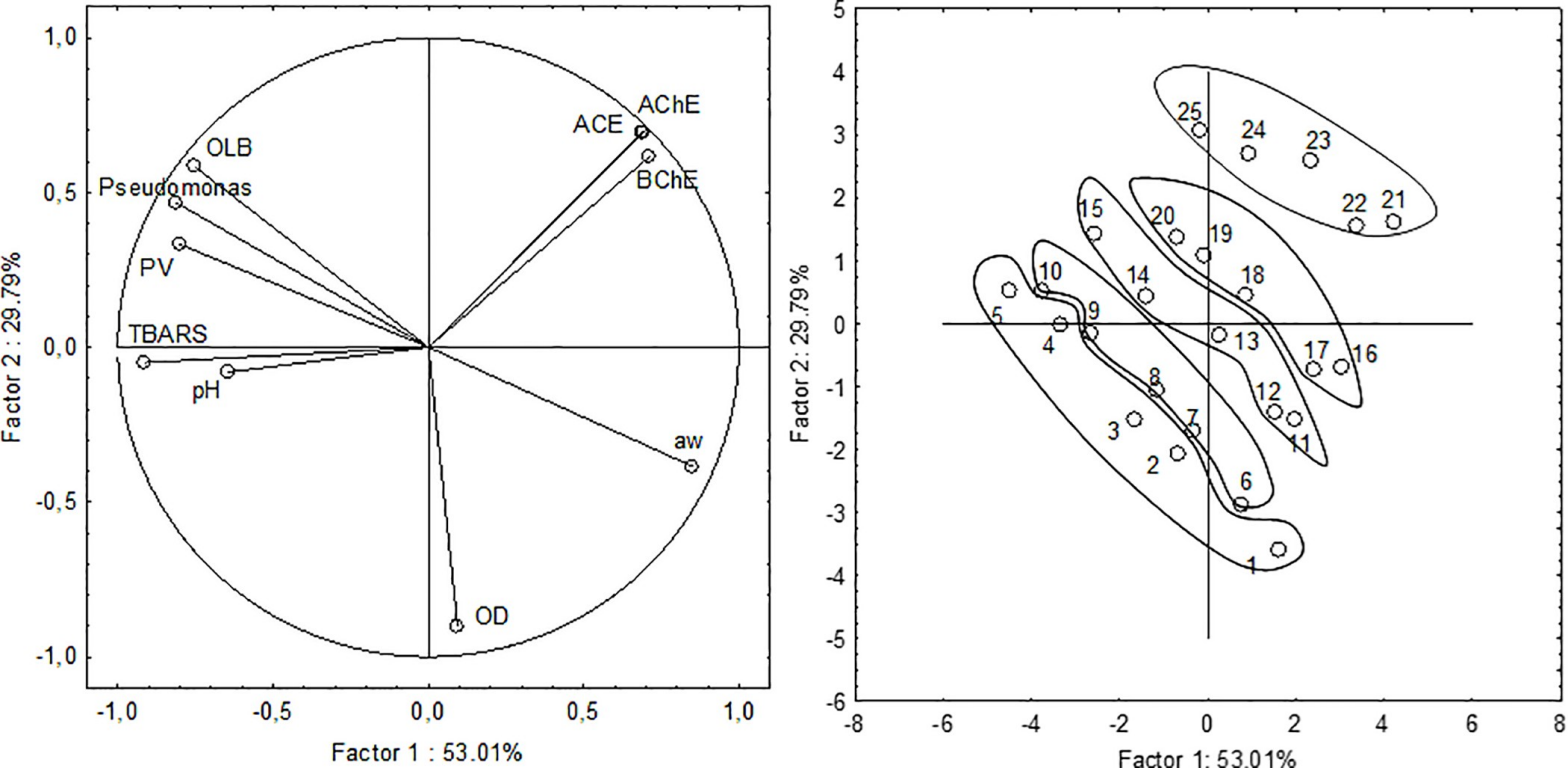
PC analysis for the tested discriminants and data point plot referring to the peroxide value (PV), TBARS, water activity (aw), total content of *Pseudomonas* and mesophilic aerobic bacteria (OLB), cholinesterase inhibitors (AChE and BChE), angiotensin-converting enzyme (ACE) and overall desirability. 1–5 –control sample at five terms of the investigations. 6–10 –sample with 20% of animal fat substituted with rapeseed oil at five experimental time points. 11–15 –sample with 20% of animal fat substituted with rapeseed oil and 0.2% of mulberry leaf extract at five experimental time points. 16–20 –sample with 20% of animal fat substituted with rapeseed oil and 0.6% of mulberry leaf extract at five experimental time points. 21–25 –sample with 20% of animal fat substituted with rapeseed oil and 1.0% of mulberry leaf extract at five experimental time points.

Principal component analysis (PCA) is used, among others, to reduce the number of variables describing phenomena, and to establish relationships between variables. It is based on determining the components that are a linear combination of the studied variables. To illustrate the assessment of the degree, to which each of the examined variables is represented by the current set of factors, two-dimensional plots were prepared that are a space projection of the tested samples onto the plane of the main components (Fig 2).

The further variable is located from the center of the circle, is better represented by the current coordinate system. The first two main factors accounted for 82.80% (factor 1 = 53.01% and factor 2 = 29.79%) of the total variability. An analysis was performed according to the planes, which were defined by the pairs of main factors, to find out which parameters were similar to each other and which differentiated experimental samples. Mutual similarities were determined based on the angle formed between two vectors of weights with the beginning at [0 0] and the ends defined by the respective values of variable weights on the considered projections. Factor 1 was found to be negatively correlated with the total number of microorganisms and bacteria of the genus *Pseudomonas*. In addition, it was noted that the following parameters had a significant contribution to the formation of the first factor: AChE and BChE and a_w_ (positive) and TBARS (negative), because their absolute weight values were the highest. Factor 2 was negatively correlated with the overall desirability of the examined sausages.

The results of the PCA analysis showed noticeable differences between the controled sample and the variant with animal fat substitution with rapeseed oil and samples containing rapeseed oil and mulberry water extract.

## 4. Discussion

White mulberry contains numerous antioxidant compounds that normalize the values of oxidative stress markers [4]. The presented work showed that mulberry contributed to a decrease in the peroxide value and TBARS index in sausages, which confirmed the benefits of using mulberry water leaf extract as a natural antioxidant.

Unstable lipid hydroxides (LOOH) completely devoid of sensory properties, are the primary oxidation products formed in the first stage of initiation. Their formation is influenced by physical factors such as high temperature during heat treatment and oxygen access, the presence of iron ions and other pro-oxidative components [26]. Even oxidatively changed fats can then have a low peroxide value. At the later stage, peroxides undergo complex and multidirectional transformations, including their decomposition, which leads to the formation of secondary oxidation products such as aldehydes and cyclic endonoxides, which are the source of malonic aldehyde [8]. These compounds, and in particular aldehydes, give the products a characteristic rancid taste and smell. In this study, where it was shown that the control sample was characterized by a lower rating of taste and smell descriptors (rancid odor and taste). The content of secondary oxidation products was indicated by the anisidine number and TBARS index, which allowed to detect the actual degree of fat oxidation [27, 28]. Changes in fat oxidation indices obtained in the current work were not linear. It was observed that the applied concentrations of mulberry leaf extract inhibited the formation of both primary and secondary oxidation products to varying degrees. This is probably the result of selective interaction of their components throughout the entire auto-oxidation process. The impact of active substances in meat products varies, and their ratio and direction depend primarily on technological parameters of the production process, fatty acid composition and initial lipid oxidation [8].

It is believed that the initial degree of microbial contamination, free water content and storage temperature are factors affecting food product deterioration. During refrigerated storage, the quality of meat and meat products deteriorates as a result of oxygen microflora development, tissue and bacterial enzyme activity, oxidation of heme pigments, lipid oxidation and surface drying due to water evaporation [29, 30]. *Pseudomonas* bacteria are part of the saprophytic microflora, which causes spoilage of meat products stored in aerobic and refrigerated conditions. They have the ability to produce extracellular enzymes at low temperature: lipases and proteinases. If the count of *Pseudomonas* exceeds 10^7^−10^9^ cfu/g, then these enzymes are responsible for the formation of irreversible changes in meat quality and an unacceptable odor [31].

According to previous reports, phenolic acids and flavonols are cholinesterase (ChE) inhibitors, as demonstrated for polyphenols present, e.g. in chia or hop seeds [16, 32]. The results of this study indicated that samples with the addition of mulberry leaf water extract exhibited high anti-ChE activity, which was also probably due to the presence of polyphenols. Studies have confirmed the presence of antioxidant compounds in white mulberry that prevent many civilization diseases such as atherosclerosis, diabetes, obesity and cancer [1, 2]. The activity of phytochemicals as cholinesterase inhibitors is widely described in the literature. Ferlemi et al. [33] demonstrated in mouse studies beneficial effects of rosemary infusion against BChE and AChE. A similar effect of rosemary leaf extract as ChE inhibitors was described in studies on rats [34]. In the literature, reports can be found on the inhibiting effect of polyphenols and plant preparations on angiotensin [35]. Research carried out in this work has confirmed that the use of mulberry leaf extract can help treat hypertension. The risk factors for atherosclerosis and related diseases, such as ischemic heart disease, myocardial infarction or stroke, i.e., hypertension, are constantly discussed in the literature. Angiotensin-converting enzyme inhibitors have a protective effect on arterial walls, manifested as the stabilization of atherosclerotic lesions. Angiotensin-converting enzyme inhibitors show not only antihypertensive but also anticoagulant, antiproliferative and nephroprotective effects [26].

## 5. Summary

For the average consumer, the product safety, sensory quality (color, texture, smell or taste of the product) is decisive when choosing food. It is perceived by the consumer in affective terms: as a degree of consumer liking and preferences. Plant extracts are a source of polyphenols, which in addition to beneficial antioxidant effects or health-promoting effects may have a bitter taste. Therefore, sensory evaluation is an important stage in the design of functional foods. This study has demonstrated that the addition of a mulberry leaf water extract not only improves the oxidative stability of fats, but also does not adversely affect the sensory values when added in the right proportion (i.e., 0.2% and 0.6%).

Mulberry leaf extract may have beneficial effects on fat stability as well as beneficial health effects in the context of prevention of neurodegenerative diseases and hypertension. The obtained sausages were characterized by favorable sensory values. These model results require further work and research that will confirm their effect in the body.

## Supporting information

S1 Data (XLSX)

S1 Graphical abstract (JPG)
