# Supplementary figures and images for: Effect of Morus alba leaf extract dose on lipid oxidation, microbiological stability, and sensory evaluation of functional liver pâtés during refrigerated storage
Source: PLoS One. 2021 Dec 23;16(12):e0260030. doi: 10.1371/journal.pone.0260030 (PMC8699953; doi:10.1371/journal.pone.0260030)

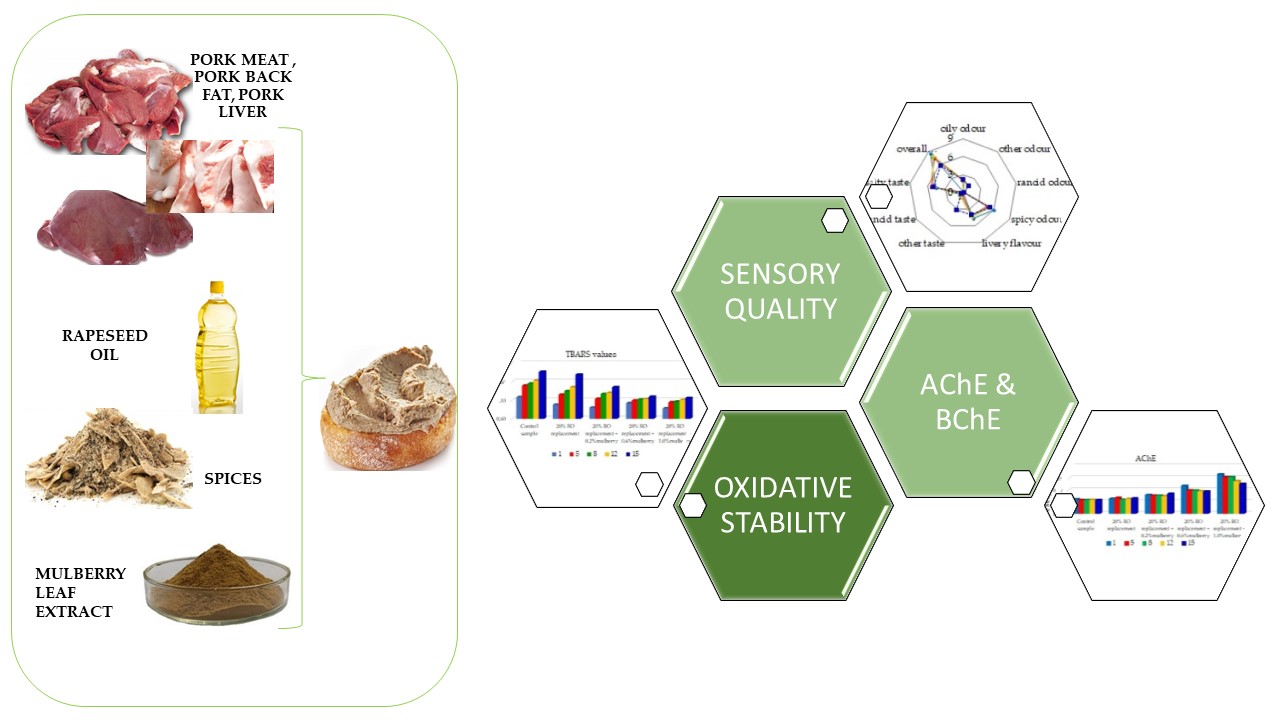

Supplement: S1 Graphical abstract — (JPG) [file pone.0260030.s002.jpg]
